# Impact of Multiple Sclerosis on Foot Health and Quality of Life: A Prospective Case-Control Investigation

**DOI:** 10.3389/ijph.2023.1605593

**Published:** 2023-02-15

**Authors:** Francisco Javier Ruiz-Sánchez, Maria do Rosário Martins, Marta Elena Losa-Iglesias, Ricardo Becerro-de-Bengoa-Vallejo, Juan Gómez-Salgado, Carlos Romero-Morales, Ana María Jiménez-Cebrián, Daniel López-López

**Affiliations:** ^1^ Research, Health and Podiatry Group, Department of Health Sciences, Faculty of Nursing and Podiatry, Industrial Campus of Ferrol, Universidade da Coruña, Ferrol, Spain; ^2^ UICISA: E, Instituto Politécnico de Viana do Castelo, Escola Superior de Saúde, Viana do Castelo, Portugal; ^3^ Faculty of Health Sciences, Universidad Rey Juan Carlos, Madrid, Spain; ^4^ Faculty of Nursing, Physiotherapy and Podiatry, Universidad Complutense de Madrid, Madrid, Spain; ^5^ Department of Sociology, Social Work and Public Health, Universidad de Huelva, Huelva, Spain; ^6^ Safety and Health Postgraduate Programme, Universidad Espíritu Santo, Guayaquil, Ecuador; ^7^ Faculty of Sport Sciences, Universidad Europea de Madrid, Madrid, Spain; ^8^ Nursing and Podiatry Department, University of Málaga, Málaga, Spain; ^9^ Instituto de Investigación Biomédica de Málaga (IBIMA), Málaga, Spain

**Keywords:** quality of life, foot care, multiple sclerosis, foot health, foot health status questionnaire

## Abstract

**Objectives:** To assess quality of life or factors related to the foot and general health and to determine the impact taking into account foot health status in people with multiple sclerosis (MS).

**Methods:** 50 subjects with MS and 50 healthy subjects were studied using the Foot Health Status Questionnaire, that is a validated and is reliable tool was used to assess foot health and quality of life. This instrument comprise four domains for evaluate the foot health (foot function, foot pain, footwear and general foot health) in the first section and for measure the general health comprise four domains (general health, physical activity, social capacity and vigor) for second section and was use for all participants.

**Results:** In both groups of the sample, 50% (*n* = 15) were men and 50% (*n* = 35) women, and the mean age in the case group was 48.04 ± 10.49 and the control group was 48.04 ± 10.45 were recruited. A statistically significant difference (*p* < 0.05) was shown for foot function, general foot health, general health, physical activity and vigor domains, stating that people with MS have a lower related to foot health (lower FHSQ scores) compared to healthy subjects who have higher FHSQ scores. There were no statistically significant differences (*p* > 0.05) for the scores of the other domains of the FHSQ (foot pain, footwear and social capacity).

**Conclusion:** Patients with MS suffer a negative impact on the quality of life related to foot health, which appears to be associated with the chronic disease.

## Introduction

Multiple sclerosis (MS) is a chronic inflammatory disease of the central nervous system (CNS), which causes large focal lesions in the white matter of the brain and spinal cord (1). Gait dysfunction is an almost ubiquitous symptom even in the early stages of the disease, several factors, such as ataxia, hypertonic muscles and deformities of the musculoskeletal system, will affect the normal contact of the plantigrade foot (2), causing a significant reduction in functional independence and wellness of patients suffering from this neurological disease (3).

In Spain, the estimated incidence of MS is 3.8 per 100,000 and the prevalence is 36–55 per 100,000, but recent studies in southern Spain state that the prevalence is higher than expected and, given the low mortality rate, can be expected to increase in the coming years (4, 5), with several studies coinciding in that the most frequent form is recurrent remitting (RR) MS, and that the female predominance has a 2 to 1 ratio (6). Women are about 2–3 times more likely to have MS, and most patients are between the ages of 20 and 50. Vitamin D deficiency is inversely related to the risk of MS and its deficiency affects 20%–25% of the population in Asia, America, Canada, Europe and Australia (7). There is strong evidence that smokers are at higher risk of MS than non-smokers (8).

It should be noted that MS patients experience a variety of deficiencies and symptoms including abnormal gait and balance, muscle weakness, spasticity, and fatigue (9); as well as neuritis, central paralysis, sensory imbalance, cognitive impairment and sleep disorders (7, 10). It is noteworthy that spasticity is one of the most prevalent chronic symptoms in MS and the one that most affects the QoL and functionality of patients. Epidemiological studies indicated that it can affect up to 80% of MS patients. Spasticity presents as increased muscle stiffness, often accompanied by spasms and impaired reflexes. In particular, the foot-related health has not been studied analytically, even thought several studied confirmed the habitual foot conditions are related with foot drop, which occurs frequently in MS due to involvement of the peroneal nerve and weak-ness of the dorsiflexor muscles, with the consequent risk of trips and falls, re-impacting the way of walking with alterations in gait patterns, forces them to perform compensatory movements such as raising the knee and turning the hips in a circular motion to lift the tip of the foot, which can cause muscle injuries, in addition to increasing fatigue (11, 12). Impaired ability to walk is a major concern for 85% of the people with MS (13, 14). Disease-modifying drugs (DMD) are the best defense to slow the progression of MS. As benefits, they cause a reduction in the frequency and severity of relapses and a reduction in the damaged areas within the brain and spinal cord, thus delaying the disability caused by this disease (15).

According to the World Health Organization, QoL is a dynamic concept based on the subjective sensation of the patient with variability over time (16). The impact of MS on QoL can be affected by numerous factors such as level of disability, type of MS, social support, education, age or employment (17–19). The most common scales that have been used to assess QoL in MS patients are the SF-36 (Short Form 36 Health Survey), the FAMS (Functional Assessment of Multiple Sclerosis) and the EDSS (Expanded Disability Status Scale) (20). Previous studies have determined that ocular alterations, lack of balance, spasticity and difficulties in walking are the factors that most affect the QoL of these patients (19, 20).

We know that MS is a chronic, progressive and disabling disease, with no expectation of cure that influences the QoL of sick people and in which the health of the feet is affected (21). However, currently QoL related to the health status of the foot in patients with MS has not been addressed in scientific studies, although clinical manifestations and conditions in the lower limbs and feet are very frequent in this pathology.

Therefore, the aim of this study was to assess foot and general health-related QoL factors by determining the impact on foot health-related QoL in a sample of MS subjects versus a group of subjects without MS.

## Methods

### Design and Sample

An observational, analytical and multicenter study of cases and controls was carried out in which subjects from MS associations in Málaga, Granada, Campillos, Marbella, Jerez and the Hospital of Ronda (Spain) were recruited from January to April 2022.

Study subjects were members of the associations of MS in Andalusia (Spain) and the healthy (controls) was recruited in a podiatric medical center that provides management of foot care in the town of Malaga (Spain). In both groups, were taken the first people with or without MS that complied the criterions for participate in this research using a convenience sampling method and they were matched to 50 assembled cases as they came forward to participate in this research.

Subjects with MS were included after being informed by the MS association that a study of the foot and wellness was to be carried out. The control subjects or healthy group were drawn from healthy people from the same locality as the cases.

The inclusion criteria were: to be between 18–88 years, of either sex, able to walk and to have authorized participation in the signing of a consent form.

The exclusion criteria of the subjects were: to have another neurodegenerative disease other than MS, cognitive impairment and severe mental disorder. Cases and controls were matched for age, gender, and BMI.

### Sample Size Calculation

A total sample calculation was designed to perform estimates of statistical power and effect size (22), as a result of the difference between two independent means (two groups) thought the G*Power 3.1.9.7 for Windows (Heinrich-Heine-Universität Düsseldorf). A two-tailed hypothesis, an effect size of 0.5, an *α* error probability of 0.05, a power (1-β error probability) of 0.6 and an allocation ratio for N2/N1 of 1 were applied. Therefore, a sample size was established consisting of 82 participants (41 per group). Finally, a total number of 100 subjects with 50 in each group participated in this research.

### Procedure

For the data collection, the research podiatrist received the participants, in similar conditions and time of day and asked them a series of questions that included anthropometric parameters (such as age, height, weight, sex), years of evolution of the disease and type of MS (in those with MS). Disability for walking and correct balance were assessed using the Berg scale. It is a clinical test of static and dynamic balance that consists of 14 simple tasks related to balance, which are scored from 0 to 4. The scores can inform us about their motor and functional capacity: initial standing group (33–39), walking start group (40–44), walking with/without technical aids (45–49), independent walking (50–54) and functional walking (55–56) (23). Subsequently, the subjects completed the Foot Health Status Questionnaire (FHSQ) Spanish version, which is a foot-specific health-related QoL measurement instrument designed and validated in Australia by Dr. Bennett et al. (24) which comprises three main sections. *Introduction* section consists of 13 questions reflecting four foot health-related domains: foot pain, foot function, footwear, and general foot health. This section has demonstrated a high degree of content, criterion, and construct validity (Cronbach *α* = 0.89–0.95) and high retest reliability (intraclass correlation coefficient = 0.74–0.92) (25), and it has been shown to be the most appropriate measure of health-related quality of life for foot health population (26). Each domain has a specific number of questions. Four regarding pain, 4 on function, 3 on footwear and 2 on general foot health. The assessment of pain and function is based on physical phenomena, the evaluation of footwear uses practical aspects related to availability and the comfort of the shoes, while the perception of the foot’s general health is based on the patients’ self-assessment of the state of their feet. Each question allows several answers and these are placed on a Likert-type ordinal scale (words or phrases corresponding to a numeric scale). The descriptors for these scales vary for each domain and the person completing the questionnaire has to choose only one response, whichever is thought to be the most appropriate. The questionnaire does not provide a global score, but rather generates an index for each domain. In order to obtain these indices, the responses are analysed by a computer program (The FHSQ, Version 1.03) which, after processing the data, gives a score ranging from 0 to 100. A 0 score represents the worst state of health for the foot and 100 is the best possible condition.

*Methods* section includes questions that reflect four general health-related domains: general health, physical activity, social capacity, and vigor. The domains and questions in this section are largely adapted from the Medical Outcomes Study 36-Item Short-Form Health Survey (27), which has been validated for use in the Australian and Spanish population (28, 29).

Finally, *Results* section collects socioeconomic status, comorbidity, service utilization and satisfaction information and their medical record.

### Ethics Procedure

To carry out our research, authorization was obtained from the Ethics Committee of the University of Malaga (CEUMA) with registration number 32–2021-H, in addition the subjects gave their permission to participate in the study by signing the informed consent form. The study was developed following the ethical principles of Clinical Research in humans (30, 31).

### Statistical Analysis

Demographic data (age, height, weight, BMI) and independent variables related with FHSQ domains levels are reported as median and interquartile range along with the range of minimum and maximum, to exception of sex that appear with frequency and percentages to describe the data.

Normality for samples of more than thirty subjects was evaluated using the Kolmogorov-Smirnov test in all variables, considering normality when *p* > 0.05 is met. In the variables that presented normality, Student’s t-test was used to find out if there was a significant difference between groups. Measurements that did not present a normal distribution were studied with the U Mann Whitney test. The FHSQ v1.03 was used to obtain QoL scores related to foot health status, where statistical significance was established at 95% CI. The gender distribution in both groups was contrasted using the Chi-square test and percentages and frequencies were applied for categorical data. All statistical analyses were carried out with SPSS v19.0 (IBM Corporation, Armonk, NY, United States).

## Results

### Descriptive Data

A total of 100 subjects between 24 and 66 years of age participated in this research. The analyzed sample included 50 subjects with MS and 50 subjects without MS; 30% (*n* = 30) were men and 70% (*n* = 70) women. The mean age was 48.04 ± 10.41. The years diagnosed were taken as it appeared in their health clinical history or data sheet of the MS association. People who suffer from the disease have an evolution of 10 ± 12 (1–33) years. Table 1 shows that the sociodemographic data of the subjects who participated in our study do not present significant differences between both groups (*p* > 0.01). After collecting data from our research, we have observed that the most used DMDs for MS were: anti-inflammatories [dimethyl fumarate (18%)], immunomodulators [interferon (26%), teriflunomide (14%), fingolimod (10%), glatiramer acetate (4%)], immunosuppressant [cladribine (8%)], monoclonal antibodies [ocrelizumab (4%), rituximab (2%)], and muscle relaxant [tizanidine (2%)]. Of the sample taken, 12% did not take any type of medication. Based on the Berg scale for the assessment of disability during walking, it was observed that 100% (*n* = 50) of the participants did not present alterations in walking and were within the functional walking group.

**TABLE 1:** Descriptive data of the sample (Spain, 2022).

Descriptive data	MS (*n* = 50)		Healthy (*n* = 50)		*p*-value*
	Mean ± SD (95%CI)	Median (IR)	Mean ± SD (95%CI)	Median (IR)	
Age (Years)	48.04 ± 10.49 (24–66)	49.00 (16.00)	48.04 ± 10.45 (24–66)	49.50 (15.00)	0.992*
Weight (kgr)	70.30 ± 13.48 (46–105)	68.50 (19.50)	72.74 ± 12.35 (47–100)	72.50 (15.80)	0.317*
Height (m)	1.67 ± 0.09 (1.50–1.83)	1.68 (0.15)	1.68 ± 0.83 (1.56–1.88)	1.65 (0.12)	0.536^†^
BMI (Kg/m2)	25.09 ± 3.99 (18.00–37.50)	23.85 (6.25)	25.73 ± 4.49 (18.40–37.20)	24.65 (5.88)	0.341^†^
Sex, m/f (%)	15/35 (30/70)		15/35 (30/70)		1.000^‡^

Abbreviations: BMI, body mass index; M, male; F, Female. In all the analyses, *p* < 0.05 (with a 5% confidence interval) was considered statistically significant. *Student’s t-test for independent samples were performed. ^†^U Mann Whitney test were utilized. ^‡^Frequencies (percentages) and chi-square (x^2^) test were utilized.

### Outcome Measurements

The domains that did not present a normal distribution were foot pain, foot function, footwear, general foot health, physical activity and social capacity (*p* < 0.05); showing a normal distribution in general health and vigor (*p* > 0.05).

In what regards the comparison of the scores obtained with the FHSQ, results appear in Table 2. These scores were higher for the non-MS group, with normalized reference values in the section of the questionnaire assessing foot pain, footwear problems, and social capacity. In the case of foot function, general foot health, physical activities, general health and vigor there is a statistically significant difference between both groups (*p* < 0.05).

**TABLE 2:** FHSQ domains levels between MS and healthy subjects (Spain, 2022).

	MS (N = 50)		Healthy (N = 50)		*p*-Value
Outcome measurements FHSQ	Mean ± SD (95% CI)	Median (IR)	Mean ± SD (95% CI)	Median (IR)	
Foot pain	73.50 ± 26.33 (6.25–100.00)	81.25 (36.72)	81.65 ± 17.70 (29.38–100.00)	85.94 (21.25)	0.291^†^
Foot function	75.13 ± 27.82 (12.50–100.00)	81.25 (43.75)	90.63 ± 13.38 (43.75–100.00)	93.75 (14.06)	0.030^†^
Footwear	44.83 ± 38.17 (0–100.00)	41.67 (77.08)	56.50 ± 33.89 (0–100.00)	58.33 (60.42)	0.119^†^
General foot health	49.75 ± 27.69 (0–100.00)	57.5 (38.13)	64.35 ± 23.92 (12.50–100.00)	60 (26.25)	0.013^†^
General health	57.00 ± 25.50 (0–100.00)	60.00 (40.00)	75.60 ± 19.60 (30.00–100.00)	80.00 (30.00)	0.001*
Physical activity	68.44 ± 29.75 (0–100.00)	80.55 (50.00)	85.89 ± 23.84 (5.56–100.00)	94.44 (16.67)	0.004^†^
Social capacity	79.25 ± 29.73 (0–100.00)	100.00 (40.62)	89.50 ± 15.84 (50.00–100.00)	100.00 (25.00)	0.366^†^
Vigor	48.50 ± 24.21 (0–100.00)	50.00 (37.50)	61.13 ± 16.62 (18.75–93.75)	62.50 (25.00)	0.001*

Abbreviations: FHSQ, foot health status questionnaire. *, Independent Student’s t -tests were employed. ^†^, the U Mann Whitney test was employed. *p*-value < 0.05 with a 95% confidence interval was considered statistically significant.

Therefore, results indicated that people with MS have a lower QoL related to foot health (lower FHSQ scores) compared to healthy subjects who have higher FHSQ scores. There were no statistically significant differences (*p* > 0.05) for the scores of the other domains of the FHSQ (foot pain, footwear and social capacity).

## Discussion

The objective of this study was to ascertain the impact that MS has on QoL related to foot health, comparing it in a group of subjects with MS with another control group of healthy subjects. This is the first analytical study to look at how MS affects foot related QoL.

According to our study, no significant difference (*p* > 0.05) was shown between domains levels between MS and healthy subject regarding foot pain, footwear and social capacity. In the domains area where there was a significant difference (*p* < 0.05) was in foot function, general foot health, physical activities, general health and vigor, where new in-depth investigations would have to be carried out to detect changes in these domains and relate it to the years diagnosed in patients with MS.

It is known that in subjects with MS, the functionality of the lower limbs is very important due to the influence of this pathology on joint mobility and muscle deficits, thus having a great impact on their psychomotor health. In recent reviews, according to the results of Stephen et al. (32), the treatment and long-term prospects of these patients have improved by introducing pharmacological therapies at an earlier stage in the disease; As Sophie et al. (33) also indicate, early diagnosis and treatment is crucial in youths, establishing a new induction therapy with better prognoses than the traditional one. Therefore, we must recognize the importance of the study of the foot in this pathology as a consequence of the conditions it causes.

In addition, different studies were focused on the assessment of QoL in patients with MS show that there is a deterioration of QoL with poor health promotion in terms of the clinical and motor symptoms where is made to the importance of physical rehabilitation programs for improvement of symptoms, as well as the inclusion of different health professionals for a better management of this pathology, resulting in an improvement in QoL (34, 35).

Furthermore, there are few studies relating QoL together with the state of foot health and general wellbeing within this pathology. For this reason, with our study we identified that subjects with MS had an affected QoL with respect to foot wellbeing, compared to subjects without MS.

Nevertheless, all our results in the group with MS are of a lower value compared to those of the group of healthy subjects, this difference being statistically significant in most of the domains analyzed, such as foot function, general foot health, general health, physical activity and vigor. A recent investigation where the wellness in relation to foot health was studied with the FHSQ questionnaire in patients suffering from chronic foot pain, coincides with ours with results that indicate that foot function, general health and physical activity are affected domains that alter and affect the patients’ QoL (36). In another similar study with Parkinson’s patients (37), it was concluded that this disease negatively affected wellness in relation to foot health, with the same domains being affected as those in our study with MS patients, both being neurological diseases.

Consistent with similar studies in subjects with poor foot wellbeing (38), foot pain (39), and joint disorders such as rheumatoid arthritis (40–42), our results also refer to the importance of physical activity as a fundamental aspect for improving QoL in subjects with MS, and in those who present deterioration.

Taking into account all of the above in relation to the affectation of QoL and foot health status, the results indicate the importance of the need to approach the treatment of this disease with a multidisciplinary team. Therefore, a correct evaluation of the state of the foot by a multidisciplinary team could be essential to reduce the increase in disabilities, neuromuscular disorders or symptoms throughout the evolution of MS.

Finally, this study has some limitations such as the walking status among the cases which may be the main sampling bias. Also, the consecutive sampling bias should be studied and a simple randomization sampling method could be more suitable for future investigations for improve that the results generalizability. Another limitation is the location of the participants where, from the entire Autonomous Community of Andalusia, only people from six geographical points wanted to participate in the study. All of this should be considered in future studies to give greater strength to the study and improve the results obtained with the different questionnaires applied in this pathology with respect to QoL and the foot.

### Conclusion

Patients with MS suffer a negative impact on the quality of life related to foot health, which appears to be associated with the chronic disease.
